# Purification and Characterization of Phenylalanine Ammonia Lyase from *Trichosporon cutaneum*

**DOI:** 10.1155/2013/670702

**Published:** 2013-09-12

**Authors:** Andrea Goldson-Barnaby, Christine H. Scaman

**Affiliations:** ^1^Food, Nutrition, and Health, University of British Columbia, 2205 East Mall, Vancouver, BC, Canada V6T 1Z4; ^2^Department of Chemistry, University of the West Indies, Kingston, Jamaica

## Abstract

*Trichosporon cutaneum* phenylalanine ammonia lyase was selected as a model to investigate the dual substrate activity of this family of enzymes. Sequencing of the PAL gene identified an extensive intron region at the N-terminus. Five amino acid residues differing from a prior report were identified. Highest Phe : Tyr activities (1.6  ± 0.3 : 0.4 ± 0.1 **μ**mol/h g wet weight) were induced by Tyr. The enzyme has a temperature optimum of 32°C and a pH optimum of 8–8.5 and shows no metal cofactor dependence. Michaelis-Menten kinetics (Phe, *K*_*m*_  5.0  ±  1.1 mM) and positive allostery (Tyr, *K*′  2.4  ±  0.6 mM, Hill coefficient 1.9 ± 0.5) were observed. Anion exchange chromatography gave a purification fold of 50 with 20% yield. The His-Gln motif (substrate selectivity switch region) indicates the enzyme's ability to act on both substrates.

## 1. Introduction

Phenylalanine ammonia lyase (PAL, EC 4.3.1.24) catalyzes the conversion of phenylalanine to *trans*-cinnamic acid, as a step in the phenylpropanoid pathway of plants and in the formation of secondary products of metabolism in some microorganisms [1, 2]. In some instances, the enzyme also converts tyrosine to *para*-hydroxycinnamic acid. These dual substrate enzymes are classified as phenylalanine/tyrosine ammonia lyases, (EC 4.3.1.25). Enzymes with a greater catalytic efficiency for tyrosine are known as tyrosine ammonia lyase (TAL, EC 4.3.1.23). There are no known genes that code for a lyase that has activity exclusively with tyrosine.

Interest in PAL is from two perspectives. First, the structural features of the enzyme responsible for its substrate specificity have not been fully elucidated. Second, a more selective and efficient TAL is of interest for industrial applications. The specificity of PAL for phenylalanine relative to tyrosine varies by over 10^6^ between biological sources, and typically, the efficiency of Phe turnover is higher than Tyr [3, 4]. An understanding of the basis for this astounding range of substrate preference is required to rationally engineer an efficient tyrosine-specific enzyme for use in the synthesis of *p*-hydroxycinnamic acid for industrial applications [5]. The microbial production of aromatic chemicals continues to increase as it allows for the use of greener technologies and renewable energy sources [6].

PAL has been extensively characterized from a wide variety of plant sources [7], but only a few microbial sources of the enzyme have been investigated [8, 9]. PAL from the yeast *Trichosporon cutaneum* (TcPAL), identified as an enzyme able to metabolize both Phe and Tyr and possessing a relatively high level of activity with tyrosine, was selected as a model to further investigate the dual substrate activity of this family of enzymes. Several novel findings were noted and are reported herein. The cloned gene was found to have a single intron region near the N-terminus of the enzyme and five amino acid residues that differed from a previous report [6]. A His-Gln motif was identified which appears to be a characteristic feature of PAL enzymes displaying dual substrate activity with tyrosine and phenylalanine. This is the first reported characterization of the TcPAL enzyme with regard to its temperature and pH optimum as well as metal dependence.

## 2. Materials and Methods

### 2.1. Cloning of the TcPAL Gene

*Escherichia coli* bacterial strains were cultured in Luria Bertani (LB, Mediatech, Herndon, VA) liquid medium. Kanamycin (50 *μ*g/mL) was added to LB media and plates when growing bacteria containing plasmids.

The yeast strain *Trichosporon cutaneum* (ATCC 58094) was purchased from the American Type Culture Collection (Manassass, VA). Shake cultures were grown aerobically in Dagley's medium at 30°C at 100 rpm with addition of tyrosine (2.0 mM) at 30°C to an OD600 of 3. Cells were pelleted by centrifugation (9000 xg, 15 min, 4°C). Genomic DNA was extracted utilizing a Qiagen DNA purification kit and utilized as template for PCR amplification.

Primer sequences were designed based on the DNA sequence of the *T. cutaneum* PAL protein. The PAL gene was PCR amplified using Phusion DNA polymerase (New England Biolabs) from *T. cutaneum* genomic DNA with the 5′ primer TcPAL- F: 5′-CGCGAATTCATGTTTATTG AGACC-3′ as the forward primer (EcoRI site underlined) and 3′ primer TcPAL- R: 5′-GAAGCTTTTAGAACATCTTGCCAAC-3′ as the reverse primer (HindIII site underlined). Control reactions were also performed. The amplified PCR fragment was purified, digested with HindIII and EcoRI, and inserted in the pET30a vector to give plasmid pET-30a (+) TcPAL. *E. coli* DH5*α* competent cells were transformed with the plasmid construct by use of heat shock treatment. Positive clones were identified by restriction analysis, and plasmid DNA was submitted for DNA sequencing.

Database comparison was performed with the BLAST search tools on the server of the National Center for Biotechnology Information, National Library of Medicine, NIH (http://www.ncbi.nlm.nih.gov/). Multiple sequence alignments were performed using ClustalW. Intron analysis was performed using GENSCAN.

### 2.2. Cell Culture and Extraction

Shake cultures of *T. cutaneum* were grown aerobically in Dagley's medium at 30°C at 100 rpm with addition of tyrosine (2.0 mM) at 30°C to an OD600 of 3. Cells were harvested by centrifugation at 23,500 xg for 15 min at 4°C. The cell pellet was resuspended in extraction buffer (50 mM Tris-HCl, pH 8.0, 1 mM EDTA, 10 mM 2-mercaptoethanol, protease inhibitor cocktail tablets EDTA free, and 1 tablet per 100 mL) in a 1 : 4 ratio (wet weight : buffer), and the cells were disrupted by vortexing with glass beads for 6 min with intermittent cooling on ice. After centrifugation, aliquots of supernatant were desalted using a PD-10 column (GE Healthcare) and used for enzyme purification. Protein concentration was determined by the Bradford assay [10].

### 2.3. Enzyme Assays

The PAL/TAL activity of enzyme extracts was measured using a spectrophotometric assay [11]. For preliminary induction studies, the enzyme activity was determined using an end point assay using Phe (40 mM) or Tyr (2 mM) in 10 mM Tris-HCl (pH 8.5) at a temperature of 37°C for 15 min. The reaction was terminated by the addition of 25% trichloroacetic acid (200 *μ*L). Samples were centrifuged at 13,000 xg for 15 min, and the absorbance was measured. All other experiments were conducted utilizing a continuous spectrophotometric assay at a temperature of 30°C for 2 min. Assays were initiated by addition of enzyme extract (200 *μ*L) to substrate solution (800 *μ*L) preequilibrated at 30°C. For kinetic analysis, solutions containing Phe (0.5 mM to 100 mM) or Tyr (0.5 mM to 10 mM) in 10 mM Tris-HCl (pH 8.0) were utilized. The same enzyme extract was used to compare the kinetic constants for PAL and TAL activities. The absorbance of *trans*-cinnamic acid was measured at 290 nm, whereas *para*-hydroxycinnamic acid was measured at 315 nm. Kinetic data was analyzed using GraphPad Statistical Software (Version 5, California) which combines biostatistics, curve fitting (nonlinear regression), and scientific graphing into a comprehensive program.

### 2.4. PAL Purification and Characterization

All purification steps were carried out at 4°C. Crude cell free extract of *T. cutaneum* from PD-10 columns was applied to a Fast Protein Liquid Chromatography anion exchange HiTrap Q Sepharose column (2.5 × 1.0 cm) pre-equilibrated with Tris-HCl buffer (0.1 M, pH 8.0). The adsorbed enzyme was eluted with a linear gradient of KCl from 0 to 0.3 M in the same buffer [12]. Fractions with PAL activity were pooled and exchanged with extraction buffer using Millipore centrifugal filter units with a 30 kDa molecular cutoff.

The temperature optimum of the enzyme was determined by performing enzyme assays at 28°C, 32°C, 36°C, and 40°C with Tris-HCl (50 mM, pH 8.0) as buffer. The pH optimum was determined by conducting enzyme assays at 32°C with Tris-HCl (50 mM) adjusted to 7.3, 7.6, 8.2, 8.6, and 8.9. Metal dependence studies were performed using the chloride salts of sodium, potassium, magnesium, and ferrous at a total salt concentration of 100 mM in Tris-HCl buffer (50 mM, pH 8.0).

## 3. Results and Discussion

### 3.1. Sequencing of the PAL Gene

The PAL gene from *T. cutaneum* was amplified from genomic DNA using PCR. A 2114 bp fragment size was generated, cloned into the pET30a vector, and submitted for sequence analysis. The gene sequence showed high similarity with other PAL enzymes in the NCBI database. Protein blast analysis revealed that the PAL shares identity with the yeast PALs *Trichosporon asahii* (70%), *Rhodosporidium toruloides* (50%), *Rhodotorula mucilaginosa* (49%), and *Rhodotorula glutinis* (48%). 

Sequencing of the TcPAL gene from two independent clones revealed five amino acid residues that differ from the sequence previously reported by Breinig et al. [6], namely, Gln 74, Ala 274, Val 298, Pro 322, and Lys 486. A comparison of our TcPAL sequence with the closely related *Trichosporon ashaii* PAL revealed that the residues at positions 74, 274, and 486 were identical (Table 1). However, the residue equivalent to 298 matched the Ala residue previously reported rather than the Val in our sequence. There was no obvious consensus for the amino acid residue corresponding to Pro 322 for the three proteins. Analysis of the *T. cutaneum* gene sequence by GENESCAN utilizing the parameter matrices for *Arabidopsis* and maize predicted the presence of an intron within the TcPAL gene sequence at position 121 through 1182 (1062 bp), which was confirmed with the translated protein sequence. 

**Figure 1:**
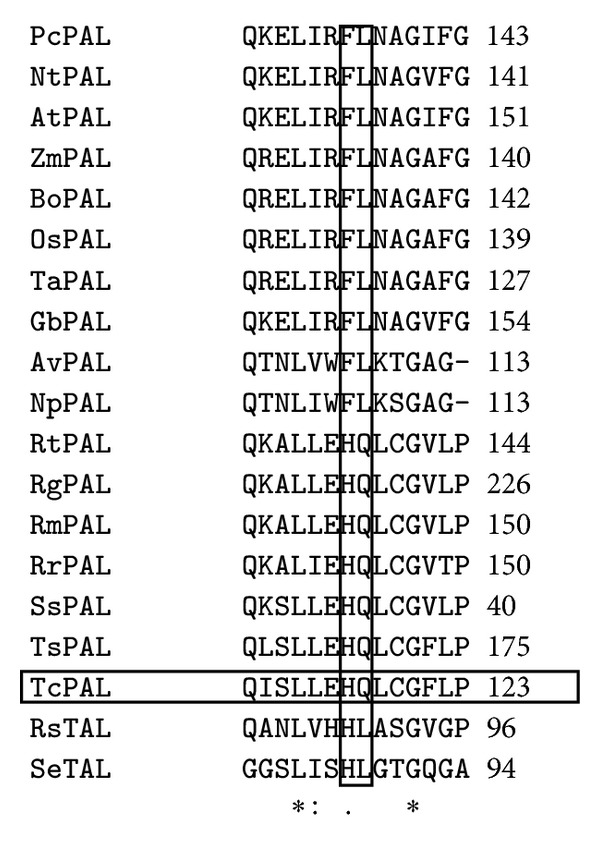
Substrate selectivity switch region (residues in bold) identified in PAL and TAL enzymes. FL is indicative of PALs, HQ for PAL/TALs, and HL for TAL enzymes. Abbreviations with Uniprot or Genbank number: Pc: *Petroselinum crispum* (P45729), Nt: *Nicotiana tabacum* (P25872), At: *Arabidopsis thaliana* (P35510), Bo: *Bambusa oldhamii* (C0LL35), Zm: *Zea mays* (B6SWA0), Os: *Oryza sativa* (A2X7F7), Ta: *Triticum aestivum* (Q43210), Gb: *Ginkgo biloba *(A7UHB6), Av: *Anabaena variabilis *(Q3M5Z3), Np: *Nostoc punctiforme* (B2J528), Rt: *Rhodosporidium toruloides *(P11544), Rg: *Rhodotorula glutinis* (G0SVG1), Rm: *Rhodotorula mucilaginosa *(P10248), Rr: *Rhodotorula graminis* (CAD23828.1), Ss: *Sporidiobolus salmonicolor* (E0YNE2), Ts: *Trichosporon asahii* (EKD03338.1), Tc: *Trichosporon cutaneum* (ABA69898.1), Rs: *Rhodobacter sphaeroides* (Q3IWB0), and Se: *Saccharothrix espanaensis* (Q2EYY5).

In the substrate selectivity switch region of PAL : TAL enzymes a His-Leu motif is characteristic of TALs, whereas Phe-Leu is characteristic of PALs [3]. In TcPAL, His-Leu was replaced by His-Gln. This motif was also observed in other microbial PAL enzymes (Figure 1). Other P/TAL enzymes with the His-Gln motif possess dual substrate activity to Phe and Tyr, with a *K*_*m*__Phe/Tyr_ ratio greater than one. Examples include *T. cutaneum* with ratios of 3.2 or 8.2 (current work, [13]), *R. toruloides*, ratio 1.6 [14], *R. glutinis,* ratio 2.3 and 1.9 [9, 15],* R. mucilaginosa,* ratio 2.0 [13], and *R. graminis,* ratio 2.9 [13]. Activity with Tyr has not been previously reported for *T. asahii*, but based on this motif, it is predicted to possess both PAL and TAL activities. In addition to the His-Gln selectivity switch region observed in the protein sequence of these enzymes, other residues surrounding this motif were conserved, specifically, Glu *His Gln* Leu Cys. Residues forming the hydrophobic substrate binding pocket described by Watts et al. [3] were also conserved (Leu 194, Leu 242, and Val 245).

### 3.2. PAL Purification and Characterization

**Figure 2:**
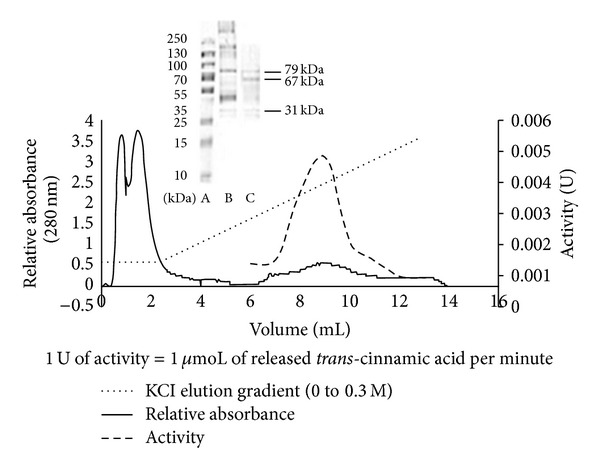
Chromatographic profile illustrating the purification of the PAL enzyme from *Trichosporon cutaneum* extract by anion exchange chromatography. Insert: SDS PAGE of *Trichosporon cutaneum* PAL enzyme. Lane A, protein ladder; B, crude extract; C, purified protein from anion exchange chromatography. *T. cutaneum *PAL at 79 kDa.

**Figure 3:**
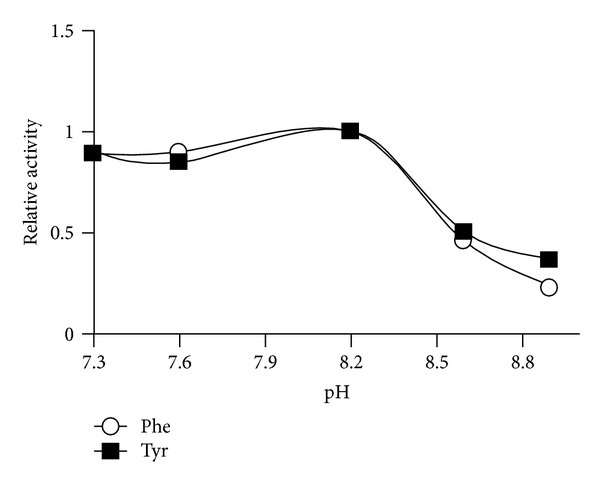
pH dependence studies on the PAL enzyme from the yeast *Trichosporon cutaneum*. A pH optimum of 8.2 was observed.

**Figure 4:**
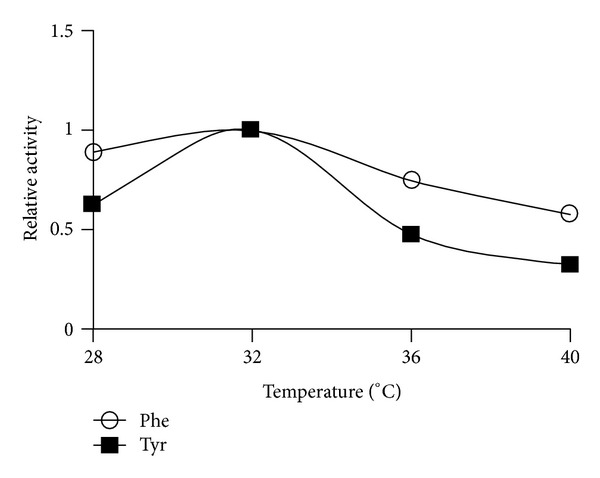
Temperature dependence studies on the PAL enzyme from the yeast *Trichosporon cutaneum*. A temperature optimum of 32°C was observed.

PAL purification usually requires multiple steps to obtain a homogenous preparation [16]. Due to the enzyme's relative instability, a rapid purification procedure is desirable. Purification of TcPAL by acid precipitation, aqueous two-phase partitioning, and anion exchange chromatography was investigated (Table 2). TcPAL was obtained with an approximate yield of 20% and 50-fold purification from the HiTrap Q Sepharose column (Figure 2). SDS PAGE of the eluted protein revealed the presence of the desired band at 79 kDa, confirmed by Western blot (not shown) and two other unidentified bands at 67 kDa and 31 kDa. PAL is typically tetrameric with four identical monomers ranging from 77 to 83 kDa [17]. This two-step procedure is rapid and efficient in comparison to the four-step procedure reported by Vannelli et al. [13] which produced a purification fold of 14 and an overall yield of 6%. Vannelli et al. [13] used a quaternized polyethyleneimine anion exchange column, resulting in a 4-fold purification. The theoretical pI of TcPAL reported by Vannelli et al. [13] was 6.3, while the pI of the enzyme as determined from isoelectric focusing analyses was 5.8. TcPAL from the present study has a theoretical pI of 6.2 because of the sequence differences we obtained, which may account for the difference observed in the behaviour of the enzyme during anion exchange chromatography. The enzyme has a pH optimum in the range of 8.0–8.5 (Figure 3) and a temperature optimum of 32°C (Figure 4) and shows no metal dependence on the chloride salts of sodium, potassium, magnesium, and ferrous. 

TcPAL exhibited the highest activity with Phe : Tyr (1.6 ± 0.3 : 0.4 ± 0.1 *μ*mol/h g wet weight) when induced by tyrosine (2 mM). Control and phenylalanine (2 mM) induced cultures possessed lower activities (Phe : Tyr 0.9 ± 0.03 : 0.2 ± 0.1 and 0.4 ± 0.2 : 0.3 ± 0.1 *μ*mol/h g wet weight, resp.), while no activity was found when 2 mM glucose was in the growth media, consistent with previous reports [17]. 

**Figure 5:**
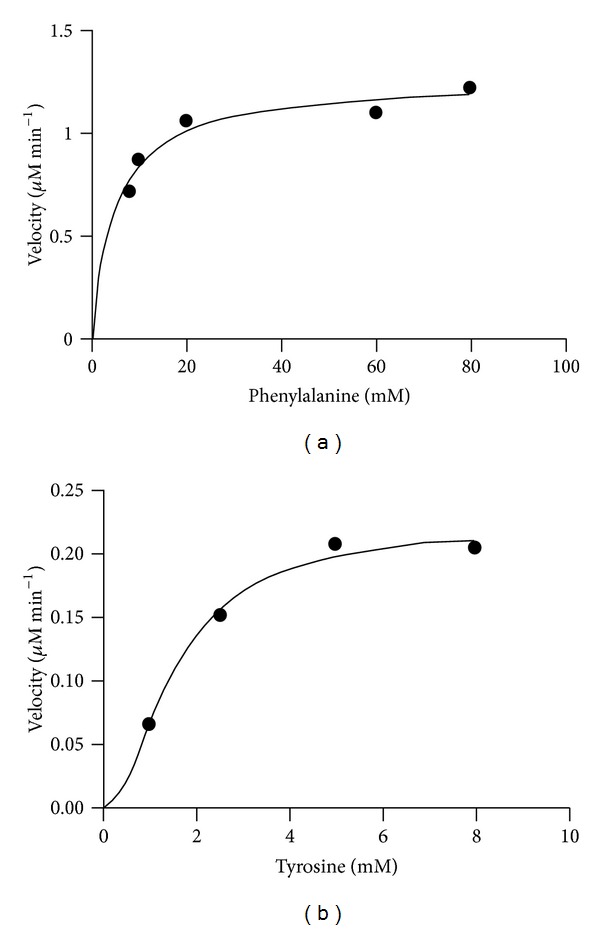
Purified *Trichosporon cutaneum* PAL kinetics. (a) Phenylalanine *K*_*m*_ 5.0 ± 1.1 mM, and *R*² = 0.93. (b) Tyrosine *K*′ = 2.4 ± 0.6 mM, *H* = 1.9 ± 0.5, *R*² = 0.99. Kinetic data was analyzed using GraphPad Statistical Software.

Purified TcPAL displayed typical Michaelis-Menten kinetics with Phe but atypical Michaelis-Menten kinetics were the best fit with Tyr (Figure 5). Vannelli et al. [13] also reported typical Michaelis Menten kinetics with Phe (*K*_*m*_ 4.9 ± 0.9 mM) and positive allostery with Tyr (*K*′ 0.6 mM, Hill coefficient 2.6 ± 0.4) comparable to the findings of this present study. Interestingly, the yeast PALs from *R. toruloides*, *R. glutinis, *and *R. mucilaginosa *with high protein sequence identity to TcPAL appear to follow Michaelis-Menten kinetics with both substrates. Positive allostery may be a characteristic feature of the *Trichosporon* species, and it is possible that *Trichosporon asahii* PAL may share similar enzyme kinetic characteristics to that of TcPAL. 

Vannelli et al. [13] reported a PAL/TAL ratio ((*V*_max⁡_/*K*_*m*_)_Phe_/(*V*_max⁡_/*K*′)_Tyr_) of 0.8, while we observed a ratio of 2.2 (Table 3). This implies that the enzyme is intrinsically a PAL and not a TAL enzyme. The His-Gln motif observed in the substrate selectivity region of the protein sequence further substantiates this claim. 

## 4. Conclusion

Five different amino acid residues not previously reported, namely, Gln 74, Ala 274, Val 298, Pro 322, and Lys 486, were identified from translation of the TcPAL gene sequence. The His-Gln motif identified in the substrate selectivity switch region of TcPAL may potentially be utilized to assist in assigning other uncharacterized microbial ammonia lyases in the genomic database as PAL-specific enzymes possessing activity with tyrosine. TcPAL exhibits greater catalytic efficiency for Phe over Tyr, although the greatest induction of enzyme activity occurred in the presence of Tyr. We confirmed that the enzyme exhibits positive allostery with Tyr, in contrast to Michaelis Menton kinetics with Phe. A two-step purification of the enzyme was achieved by applying a crude cell extract on anion exchange chromatography (20% yield, 50-fold purification). 

## Figures and Tables

**Table 1:** Amino acids reported for *Trichosporon cutaneum* PAL and equivalent residues identified in *T. ashaii* P/TAL^a^.

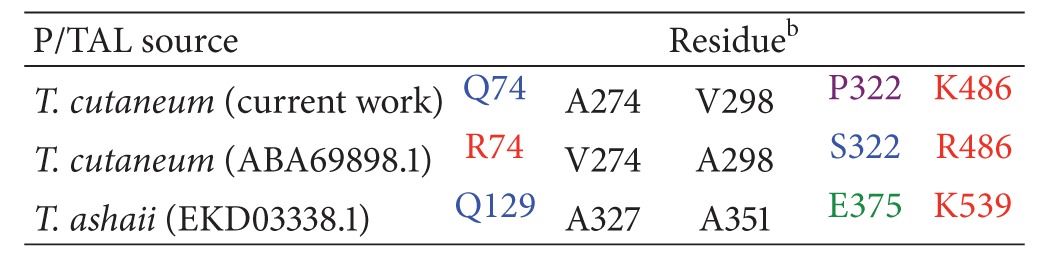

^a^Genbank number in brackets.

^b^Blue: polar uncharged residues; red: positively charged; green: negatively charged; black: polar; purple: proline.

**Table 2:** Purification of the PAL enzyme from *Trichosporon cutaneum* ^a^.

Method	Total activity^b^ (U)	Total protein (mg)	Specific activity (U/mg)	Purification fold	Yield (%)
Anion exchange					
Crude	0.05 ± 0.01	0.38 ± 0.09	0.15 ± 0.00	—	100
Post	0.01 ± 0.00	0.001 ± 0.00	7.39 ± 0.73	49.6	20
Acid precipitation					
Crude	0.16 ± 0.03	1.04 ± 0.74	0.20 ± 0.10	—	100
Post	0.15 ± 0.04	0.61 ± 0.28	0.26 ± 0.06	1.3	94
Aqueous two-phase^c^					
Crude	0.07 ± 0.03	0.39 ± 0.14	0.21 ± 0.05	—	100
PEG/Na_2_SO_4_	0.02 ± 0.00	0.08 ± 0.00	0.33 ± 0.03	1.5	28
PEG/(NH_4_)_2_SO_4_	0.02 ± 0.00	0.10 ± 0.08	0.10 ± 0.04	0.5	28
PEG/ Na_2_CO_3_	0.01 ± 0.00	0.31 ± 0.04	0.02 ± 0.01	0.1	14

^a^Results are the average ± range of two independent trials. Crude samples are cell culture extracts obtained from a PD-10 column. ^b^1 U of activity = 1 *μ*moL of released *tran-*cinnamic acid per min. ^c^For aqueous two-phase partitioning, the same crude enzyme extraction was used for each of the PEG/salt systems.

**Table 3:** Kinetic parameters for purified extracts of the PAL enzyme from *Trichosporon cutaneum*.

Trial	Phenylalanine	Phenylalanine	Phenylalanine	Tyrosine	Tyrosine	Tyrosine	PAL/TAL ratio
	*V*_max⁡_ (*μ*M min^−1^)	*K*_*m*_ (mM)	*V*_max⁡_/*K*_*m*_	*V*_max⁡_ (*μ*M min^−1^)	*K*′ (mM)	*V*_max⁡_/*K*′	
1	1.3 ± 0.1	5.0 ± 1.1	0.3	0.2 ± 0.02	2.4 ± 0.6	0.1	3.0
2	4.7 ± 0.6	5.7 ± 3.0	0.8	1.0 ± 0.1	2.0 ± 0.4	0.5	1.6
3	7.2 ± 0.8	4.2 ± 2.0	1.7	0.8 ± 0.1	0.9 ± 0.4	0.9	1.9

For each trial, activity assays for Phe and Tyr were performed on the same enzyme extract. Extracts were obtained from anion exchange chromatography.
